# Risk factors for central lymph node metastasis in papillary thyroid microcarcinoma: a simplified risk scoring system

**DOI:** 10.3389/fendo.2026.1833331

**Published:** 2026-09-07

**Authors:** Shuo Liu, Jing Zhang

**Affiliations:** Department of General Surgery, Tianyou Hospital Affiliated to Wuhan University of Science and Technology, Wuhan, China

**Keywords:** central lymph node metastasis, papillary thyroid microcarcinoma, preoperative prediction, prophylactic central neck dissection, risk scoring system

## Abstract

**Background:**

Central lymph node metastasis (CLNM) occurs in 20–50% of papillary thyroid microcarcinoma (PTMC) patients and influences surgical decision-making. Existing predictive models require complex calculations or specialized tools, limiting clinical utility. This study aimed to develop a simplified risk scoring system for preoperative CLNM prediction using readily available clinical variables.

**Methods:**

This retrospective cohort study included 486 PTMC patients who underwent thyroid surgery with prophylactic central neck dissection between January 2022 and January 2026. Multivariate logistic regression identified independent predictors of CLNM. A simplified risk scoring system was developed by multiplying regression coefficients (β) by 2 and rounding to integers. Internal validation was performed using bootstrap resampling (1000 iterations).

**Results:**

Four independent predictors were identified: male gender (β=0.52, adjusted OR 1.68, 95% CI 1.12–2.53), tumor size >5 mm (β=0.76, adjusted OR 2.14, 95% CI 1.45–3.16), multifocality (β=0.64, adjusted OR 1.89, 95% CI 1.23–2.91), and extrathyroidal extension (β=1.02, adjusted OR 2.76, 95% CI 1.68–4.53). The scoring system assigned 1 point each for male gender and multifocality, and 2 points each for tumor size >5 mm and extrathyroidal extension (total score 0–6). The apparent AUC was 0.742 (95% CI 0.698–0.786), with optimism-corrected AUC of 0.738. Risk stratification demonstrated progressive CLNM rates: very low-risk (score 0, 0%), low-risk (score 1, 22.3%), intermediate-risk (scores 2–3, 34.6%), and high-risk (scores 4–6, 62.2%) (p for trend <0.001).

**Conclusions:**

This simplified four-variable scoring system effectively stratifies CLNM risk in PTMC using preoperatively available data. Patients with score ≤1 may be considered for less extensive surgery or active surveillance, while those with score ≥4 should be considered for prophylactic central neck dissection. This practical tool enables immediate bedside risk assessment without specialized software, though external validation is warranted.

## 1 Introduction

Papillary thyroid carcinoma (PTC) is the most common endocrine malignancy, accounting for approximately 85% of all thyroid cancers (1). Papillary thyroid microcarcinoma (PTMC), defined as PTC with maximum tumor diameter ≤10 mm, has become increasingly prevalent with widespread ultrasound screening, now representing the majority of newly diagnosed thyroid cancers in many regions (2, 3).

Despite excellent prognosis, PTMC can exhibit aggressive biological behavior. Central lymph node metastasis (CLNM)—metastatic involvement of level VI lymph nodes—occurs in 20-50% of patients and is associated with increased locoregional recurrence risk and potential need for reoperation (4, 5). The optimal surgical management of the central neck in clinically node-negative PTMC remains controversial. Guideline recommendations have evolved considerably. The 2015 American Thyroid Association (ATA) guidelines suggested selective prophylactic central neck dissection (pCND) based on risk factors (6), whereas the updated 2025 ATA guidelines now strongly recommend against routine pCND for most small, clinically node-negative PTCs, while conditionally permitting selective dissection in patients with advanced primary tumors or when nodal information will guide subsequent therapy (7). This shift—from selective dissection toward risk-adapted, individualized decision-making—underscores the need for practical preoperative risk stratification tools.

Accurate preoperative risk stratification is therefore essential to guide surgical decision-making. Low-risk patients may be candidates for less extensive surgery or active surveillance, whereas high-risk patients benefit from pCND to achieve complete oncologic resection at the initial operation (8, 9). Although previous studies have identified relevant clinical risk factors for CLNM (10), existing predictive models present significant barriers to clinical implementation. Nomograms, while statistically robust, require graphical calculation or online tools that interrupt clinical workflow. Machine learning models achieve superior discrimination, but function as “black boxes” demanding specialized software and technical expertise.

Therefore, this study aimed to identify independent preoperative predictors of CLNM and develop a simplified risk scoring system using readily available clinical variables. By enabling immediate risk calculation without specialized tools, this system seeks to facilitate individualized surgical planning and support selective application of pCND in accordance with contemporary precision medicine principles.

## 2 Materials and methods

### 2.1 Study design and participants

This retrospective cohort study included consecutive PTMC patients who underwent thyroid surgery with pCND at our hospital between January 2022 and January 2026. The study was approved by the Institutional Review Board (approval number: TY-IEC2026-059). Informed consent was waived due to retrospective design.

Inclusion Criteria: 1) Histopathologically confirmed PTMC (maximum tumor diameter ≤10 mm); 2) Primary surgery with total thyroidectomy or lobectomy plus isthmusectomy; 3) pCND (level VI) performed; 4) Complete clinicopathological data available; 5) node-negative status preoperatively (no clinically suspected central lymph node metastasis).

Exclusion criteria: 1) Previous thyroid surgery, radiotherapy, or radiofrequency ablation; 2) Distant metastasis at presentation; 3) Concurrent other malignancies within 5 years; 4) Familial thyroid carcinoma; 4) Incomplete medical records.

### 2.2 Data collection

All data were retrospectively extracted from electronic medical records, surgical databases, and pathology reports by two independent researchers using a standardized data collection form, with discrepancies resolved by consensus or consultation with a third senior investigator. Clinical variables included age at surgery, sex, body mass index, history of Hashimoto’s thyroiditis diagnosed by preoperative serology and/or cytology, with histopathological confirmation from surgical specimens, and preoperative laboratory values including serum TSH and thyroglobulin levels measured within 30 days before surgery. Ultrasonographic assessments were performed by board-certified radiologists with at least 5 years of experience in thyroid imaging using high-frequency linear transducers (7–15 MHz), with all examinations completed within 30 days prior to surgery. The following sonographic features of the index lesion were recorded from archived images by two radiologists blinded to final pathology: maximum tumor diameter measured in three orthogonal planes, multifocality defined as two or more distinct foci of PTMC within the thyroid gland, tumor location categorized as upper, middle, or lower pole, isthmus, or diffuse involvement, extrathyroidal extension on ultrasonography, classified as suspicious (loss of capsular contour without definite invasion of adjacent structures) or gross (definite invasion of adjacent structures), capsular invasion indicated by tumor abutment with irregular margin, microcalcifications defined as punctate echogenic foci without posterior acoustic shadowing, and taller-than-wide shape determined when the anteroposterior diameter exceeded the transverse diameter on transverse view. Inter-rater reliability for ultrasound variables was assessed in a random subset of 50 patients, with Cohen’s kappa values exceeding 0.75 for all categorical variables. Pathological variables were obtained from standardized histopathology reports reviewed by two specialized endocrine pathologists blinded to clinical and imaging findings, including histological subtype, lymphovascular invasion confirmed by immunohistochemistry when equivocal, total number of central lymph nodes harvested from level VI regions, and number of metastatic lymph nodes with histologically confirmed PTC.

### 2.3 Surgical and pathological procedures

All surgical procedures were performed by board-certified endocrine surgeons with at least 10 years of experience in thyroid surgery, following the 2015 American Thyroid Association management guidelines. Total thyroidectomy or lobectomy plus isthmusectomy was performed based on preoperative assessment of tumor multifocality and patient preference, with the extent of thyroid resection determined by intraoperative frozen section examination when indicated. pCND was routinely performed in all patients, encompassing systematic removal of prelaryngeal (Delphian), pretracheal, and bilateral paratracheal lymph nodes (level VI). The surgical technique involved careful identification and preservation of the recurrent laryngeal nerve and parathyroid glands, with parathyroid autotransplantation performed when devascularization was unavoidable. The central neck dissection specimen was submitted as separate packets from each subregion, clearly labeled for pathological orientation. All surgical specimens were processed according to standardized institutional protocols within 24 hours of resection. The thyroid specimen was inked for surgical margin assessment, serially sectioned at 2–3 mm intervals, and entirely submitted for histological examination to ensure accurate measurement of maximum tumor diameter and detection of multifocal lesions. Lymph nodes were carefully dissected from adipose tissue, with each node bisected and entirely embedded. Hematoxylin and eosin staining was performed on 4-micrometer sections, with immunohistochemical staining for thyroglobulin and cytokeratin-19 used to confirm metastatic carcinoma in morphologically equivocal cases. Pathological assessment documented maximum tumor dimension, histological subtype according to WHO classification, presence and extent of extrathyroidal extension classified as microscopic or gross, capsular invasion, multifocality with number and location of separate foci, lymphovascular invasion, and central lymph node status including total number of nodes harvested and number of metastatic nodes with extranodal extension when present. All pathological reviews were conducted by two specialized endocrine pathologists with at least 8 years of experience, with consensus achieved for discordant cases through multihead microscope review.

### 2.4 Risk scoring system development

Based on multivariate logistic regression coefficients (β), we developed a simplified risk scoring system for preoperative prediction of central lymph node metastasis. The scoring system was constructed using the following method: To preserve relative prognostic importance while maintaining clinical simplicity, we multiplied each β coefficient by 2 and rounded to the nearest integer. This approach yielded 1 point each for male gender (β=0.52×2 = 1.04→1) and multifocality (β=0.64×2 = 1.28→1), and 2 points each for tumor size >5 mm (β=0.76×2 = 1.52→2) and extrathyroidal extension (β=1.02×2 = 2.04→2), with a total score ranging from 0 to 6 points. This approach preserves the relative prognostic importance of each variable while maintaining clinical simplicity. To validate this choice, we compared the performance of scoring systems derived from alternative scaling factors (×1, ×3, and ×4). The ×2 scaling was selected as it provided: (1) a practical score range (0–6 points) facilitating three risk tiers; (2) preservation of relative variable importance (ETE highest, male/multifocality lowest); and (3) optimal discrimination compared to simpler or more complex weighting schemes (**Supplementary Table 1**).

The final scoring system comprised four binary variables with assigned points as follows: male gender (β=0.52, 1 point), tumor size >5 mm (β=0.76, 2 points), multifocality (β=0.64, 1 point), and extrathyroidal extension (β=1.02, 2 points). The total score ranged from 0 to 6 points. Risk stratification categories were predefined based on score distribution and clinical utility: low-risk (0–1 points), intermediate-risk (2–3 points), and high-risk (4–6 points). Cutoff points were selected to maximize clinical applicability, balancing sensitivity for detecting CLNM against specificity to avoid overtreatment.

### 2.5 Model validation

Internal validation was performed using bootstrap resampling with 1000 iterations to estimate optimism-corrected performance metrics and assess model stability. In each bootstrap sample, the logistic regression model was refitted and evaluated on the corresponding out-of-bag sample. The apparent area under the receiver operating characteristic curve (AUC) was calculated from the original cohort, and the optimism was estimated as the mean difference between bootstrap performance and out-of-bag performance across all resamples. The optimism-corrected AUC was obtained by subtracting average optimism from apparent AUC. Ninety-five percent confidence intervals for all performance metrics were derived using the bootstrap percentile method.

Discrimination was assessed using receiver operating characteristic (ROC) curve analysis, with calculation of AUC and 95% confidence intervals. The Youden index (sensitivity + specificity - 1) was employed to determine optimal cutoff points for risk stratification, with alternative cutoffs evaluated for clinical scenarios prioritizing either sensitive detection of CLNM (to minimize missed metastases) or specific confirmation of high-risk status (to minimize false-positive over-treatment). Model calibration was assessed visually by plotting predicted versus observed probabilities across risk score categories.

Sensitivity analyses were conducted to evaluate scoring system robustness across clinically relevant subgroups and alternative definitions: (1) excluding follicular variant papillary thyroid carcinoma to assess histology-specific performance; (2) excluding patients with Hashimoto’s thyroiditis to evaluate autoimmune confounding effects; (3) repeating analyses using alternative tumor size cutoffs (4 mm, 6 mm, and 8 mm) to validate the >5 mm threshold selection. Each sensitivity analysis included recalculation of AUC, risk group-specific CLNM rates, and 95% confidence intervals. These exclusions were performed separately (not simultaneously) to evaluate the independent effect of each condition on model performance.

### 2.6 Sample size and statistical analysis

Based on 10 events per variable (EPV) rule, expecting 4–6 predictors with CLNM prevalence ~30%, a minimum of 200 patients with complete data was required. To account for an anticipated 20% exclusion rate due to incomplete records or ineligible criteria, we planned to screen approximately 250 consecutive patients. However, given the institutional volume of thyroid surgery, we ultimately screened 612 consecutive patients to maximize statistical power and ensure robust subgroup analyses, yielding 486 patients (20.6% exclusion rate) for the final analysis. Variables with <5% missing data were analyzed using complete case analysis. Variables exceeding 5% missing data were excluded from primary analysis.

Continuous variables were expressed as mean ± standard deviation (normal distribution) or median with interquartile range (non-normal distribution), compared using independent t-test or Mann-Whitney U test as appropriate. Categorical variables were expressed as frequencies and percentages, compared using Chi-square test or Fisher’s exact test. Inter-rater reliability for ultrasound categorical variables was assessed using Cohen’s kappa in a random subset of 50 patients. Univariate logistic regression was performed to identify potential risk factors, with variables showing p<0.10 or established clinical relevance selected for multivariate analysis. Multicollinearity was assessed using variance inflation factors (VIF), with variables showing VIF >5 excluded. Multivariate logistic regression was conducted using backward stepwise selection with likelihood ratio test (entry criterion p<0.10, stay criterion p<0.05). Adjusted odds ratios (OR) with 95% confidence intervals were calculated for all independent predictors. The final model fit was assessed using the likelihood ratio test. For risk group comparisons involving zero events (CLNM rate 0% in score 0 group), conventional logistic regression yields infinite odds ratios. We applied Firth’s penalized likelihood method (logistf package in R) to obtain finite, bias-corrected estimates and profile likelihood confidence intervals. All statistical analyses were performed using R version 4.2.1. Two-sided p-values <0.05 were considered statistically significant.

## 3 Results

### 3.1 Patient characteristics

During the study period, 612 patients underwent thyroid surgery for PTMC with pCND. Of these, 126 patients were excluded: 34 for previous thyroid surgery or radiofrequency ablation, 18 for distant metastasis at presentation, 22 for concurrent malignancies within 5 years, 8 for familial thyroid carcinoma, and 44 for incomplete medical records or missing key variables (>5% missing data). A total of 486 patients were included in the final analysis. Baseline characteristics are shown in **Table 1**.

**Table 1:** Clinicopathological characteristics of PTMC patients.

Characteristics	Total (n=486)	CLNM (-) (n=344)	CLNM (+) (n=142)	P-value
Age (years)	48.5 ± 12.3	49.8 ± 12.4	45.2 ± 11.8	0.032
Age <45 years	196 (40.3%)	128 (37.2%)	68 (47.9%)	0.021
Sex				
Male	112 (23.0%)	68 (19.8%)	44 (31.0%)	0.018
Female	374 (77.0%)	276 (80.2%)	98 (69.0%)	
Tumor size (mm)	6.8 ± 2.1	6.4 ± 2.0	7.6 ± 2.0	<0.001
Tumor size >5 mm	276 (56.8%)	178 (51.7%)	98 (69.0%)	0.001
Multifocality	138 (28.4%)	86 (25.0%)	52 (36.6%)	0.004
Extrathyroidal extension	102 (21.0%)	48 (14.0%)	54 (38.0%)	<0.001
Sonographically suspected	90 (18.5%)	48 (14.0%)	42 (29.6%)	
Gross	12 (2.5%)	0 (0%)	12 (8.5%)	
Capsular invasion	126 (25.9%)	78 (22.7%)	48 (33.8%)	0.002
Hashimoto’s thyroiditis	142 (29.2%)	102 (29.7%)	40 (28.2%)	0.742

CLNM was detected in 142 patients (29.2%). Mean number of central lymph nodes harvested was 6.2 ± 3.1. Median number of metastatic nodes was 2 (IQR 1-4).

Among included patients, individual variables had missing rates <5%: tumor location (n=12, 2.5%), TSH level (n=8, 1.6%), and body mass index (n=15, 3.1%). Complete case analysis was applied, with no patients excluded solely for variable-level missing data.

### 3.2 Univariate and multivariate analysis

Univariate logistic regression was performed for all collected variables. TSH level, body mass index, tumor location, thyroglobulin level, and taller-than-wide shape showed no significant association with CLNM (p>0.10) and lacked established clinical relevance, and were therefore not selected for multivariate analysis. Hashimoto’s thyroiditis showed no association (p=0.742) and was similarly excluded. Seven variables showed p<0.10: age <45 years, male gender, tumor size >5 mm, multifocality, extrathyroidal extension, capsular invasion, and microcalcifications. However, lymphovascular invasion was detected in only 28 patients (5.8%) with high inter-observer variability (kappa=0.68), and was excluded from multivariate analysis due to insufficient events and unreliable assessment. Multicollinearity assessment showed all VIF values <2.0. Among the six remaining candidate variables entered into multivariate analysis, microcalcifications was excluded due to collinearity with extrathyroidal extension (correlation coefficient 0.42). Age <45 years (p=0.089) and capsular invasion (p=0.112) did not reach statistical significance using backward stepwise elimination. The final model retained four independent risk factors (**Table 2**).

**Table 2:** Multivariate logistic regression analysis of risk factors for CLNM.

Risk factor	β coefficient	Adjusted OR	95% CI	P-value
Male gender	0.52	1.68	1.12-2.53	0.013
Tumor size >5 mm	0.76	2.14	1.45-3.16	<0.001
Multifocality	0.64	1.89	1.23-2.91	0.004
Extrathyroidal extension	1.02	2.76	1.68-4.53	<0.001

### 3.3 Development of risk scoring system

The final simplified risk scoring system comprised four independent predictors with assigned points as follows: male gender (β=0.52, 1 point), tumor size >5 mm (β=0.76, 2 points), multifocality (β=0.64, 1 point), and extrathyroidal extension (β=1.02, 2 points), yielding a total score ranging from 0 to 6 points. Points were derived by multiplying each β coefficient by 2 and rounding to the nearest integer, preserving relative prognostic weights while enabling immediate mental calculation (**Table 3**). Extrathyroidal extension, as the strongest predictor (β=1.02, adjusted OR 2.76), carried the highest weight. The scoring system demonstrated a progressive increase in CLNM risk with higher scores, with rates of 0% for score 0 (n=86), 22.3% for score 1 (n=112), 31.4% for score 2 (n=102), 37.5% for score 3 (n=112), and 62.2% for scores 4–6 combined (n=74). Individual score distributions within the high-risk group were: score 4 (n=32, 50.0% CLNM), score 5 (n=24, 58.3% CLNM), and score 6 (n=18, 77.8% CLNM).

**Table 3:** Detailed risk scoring system development.

Risk factor	β coefficient	Adjusted OR (95% CI)	P-value	β×2*	Final points
Male gender	0.52	1.68 (1.12-2.53)	0.013	1.04	1
Tumor size >5 mm	0.76	2.14 (1.45-3.16)	<0.001	1.52	2
Multifocality	0.64	1.89 (1.23-2.91)	0.004	1.28	1
Extrathyroidal extension	1.02	2.76 (1.68-4.53)	<0.001	2.04	2

*Points were derived by multiplying each β coefficient by 2 and rounding to the nearest integer.

### 3.4 ROC analysis and clinical cutoff points

**Figure 1:**
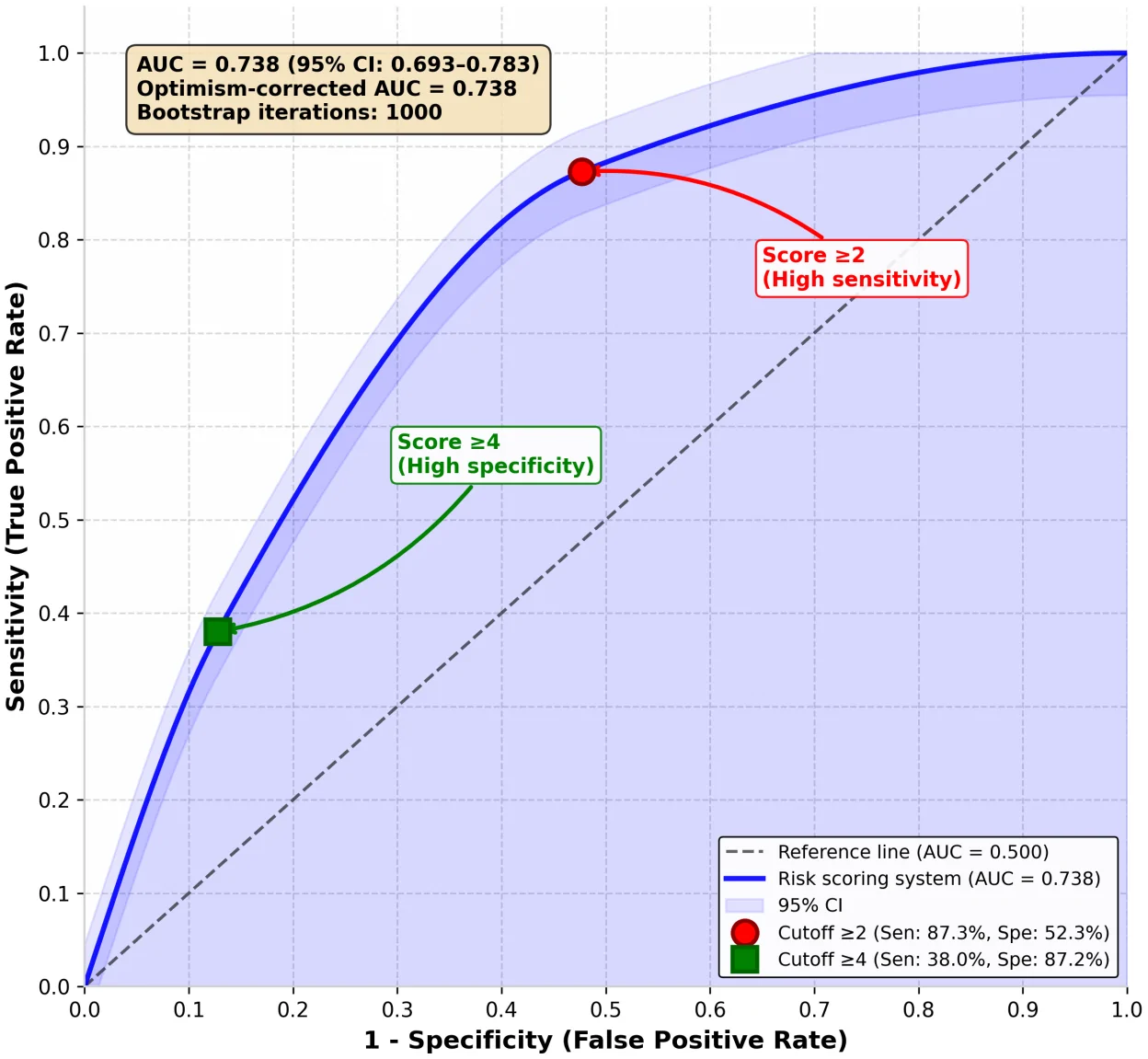
Receiver operating characteristic (ROC) curve for central lymph node metastasis prediction.

Receiver operating characteristic curve analysis demonstrated good discriminative ability with an apparent AUC of 0.742 (95% CI: 0.698-0.786) (**Figure 1**). Bootstrap resampling with 1000 iterations confirmed minimal overfitting, yielding an optimism-corrected AUC of 0.738 (95% CI: 0.693-0.783).

For optimal cutoff determination, two clinically relevant thresholds were identified. A cutoff of score ≥2 (combining intermediate- and high-risk patients) provided high sensitivity (87.3%) with moderate specificity (52.3%), prioritizing CLNM detection when comprehensive disease control is paramount. Conversely, a cutoff of score ≥4 (high-risk only) offered high specificity (87.2%) with moderate sensitivity (38.0%), prioritizing confirmation of high-risk status when avoiding overtreatment is the primary goal. These complementary thresholds allow flexible application according to specific clinical contexts and patient preferences.

### 3.5 Risk stratification

Patients were stratified into four risk categories based on score distribution and clinical utility: very low-risk (score 0), low-risk (score 1), intermediate-risk (scores 2-3), and high-risk (scores 4-6). The Cochran-Armitage test for trend was highly significant (p<0.001), demonstrating a progressive increase in CLNM risk across risk categories (**Table 4**). Notably, no CLNM was observed in the very low-risk group (score 0, n=86), while the low-risk group (score 1, n=112) had a CLNM rate of 22.3%, highlighting the distinct prognostic profiles between these subgroups. The odds ratio for CLNM in high-risk versus low-risk group was 5.6 (95% CI: 3.2-9.8). Using Firth’s penalized likelihood method to account for zero events in the very low-risk group, the odds ratio for high-risk versus very low-risk was 12.5 (95% CI: 5.2-29.7). The absolute risk difference between high-risk and very low-risk groups was 62.2 percentage points (95% CI: 50.1-73.2%).

**Table 4:** Risk stratification and CLNM rates.

Risk group	Score	n (%)	CLNM rate	95% CI
Very low-risk	0	86 (17.7%)	0%	0-4.2%
Low-risk	1	112 (23.0%)	22.3%	15.2-30.8%
Intermediate-risk	2-3	214 (44.0%)	34.6%	28.4-41.2%
High-risk	4-6	74 (15.2%)	62.2%	50.1-73.2%

Cochran-Armitage test for trend p<0.001; ^a^OR for high-risk vs. very low-risk: 12.5 (95% CI: 5.2-29.7) calculated using Firth’s penalized likelihood method due to zero events in very low-risk group; OR for high-risk vs. low-risk: 5.6 (95% CI: 3.2-9.8). Firth’s penalized likelihood method applied to account for zero events in very low-risk group (0/86, 0% CLNM). Conventional maximum likelihood estimate is undefined.

### 3.6 Model robustness and threshold optimization

The >5 mm tumor size threshold demonstrated optimal discrimination (AUC 0.738) compared to alternative cutoffs of 4 mm (AUC 0.712), 6 mm (AUC 0.721), and 8 mm (AUC 0.698), supporting its selection as the clinically optimal threshold (**Table 5**). The >5 mm cutoff minimized the low-risk CLNM rate (12.6%) while maximizing the high-risk CLNM rate (62.2%), with the highest Youden index (0.173).

**Table 5:** Model robustness across tumor size thresholds and clinically relevant subgroups.

Analysis	n	AUC (95% CI)	Low-risk CLNM rate	High-risk CLNM rate
Tumor size threshold				
>4 mm	486	0.712 (0.666-0.758)	18.3%	55.4%
>5 mm (final)	486	0.738 (0.693-0.783)	12.6%	62.2%
>6 mm	486	0.721 (0.675-0.767)	15.4%	58.6%
>8 mm	486	0.698 (0.651-0.745)	21.7%	52.1%
Sensitivity analyses				
Excluding follicular variant PTC	418	0.751 (0.704-0.798)	11.8%	64.5%
Excluding Hashimoto’s thyroiditis	344	0.745 (0.696-0.794)	13.2%	61.8%
Age <55 years	312	0.741 (0.691-0.791)	13.4%	63.8%
Age ≥55 years	174	0.728 (0.662-0.794)	10.5%	58.3%
Male patients	112	0.752 (0.683-0.821)	14.3%	65.2%
Female patients	374	0.731 (0.679-0.783)	12.1%	60.8%

Sensitivity analyses confirmed model robustness across clinically relevant scenarios. Excluding follicular variant PTC (n=68) yielded consistent performance (AUC 0.751, 95% CI: 0.704-0.798). Exclusion of Hashimoto’s thyroiditis patients (n=142) also showed consistent results (AUC 0.745, 95% CI: 0.696-0.794). Stratified analyses by age (<55 vs. ≥55 years) and gender showed comparable discrimination (AUC range 0.728–0.752), further supporting generalizability.

The >5 mm threshold demonstrated optimal discrimination with the highest Youden index (0.173). Sensitivity and subgroup analyses confirmed consistent model performance across diverse patient populations (AUC range 0.728–0.752).

## 4 Discussion

This study identified four independent predictors of central lymph node metastasis in papillary thyroid microcarcinoma: male gender, tumor size >5 mm, multifocality, and extrathyroidal extension. The simplified risk scoring system demonstrated good discrimination (AUC 0.738) and clear risk stratification, with CLNM rates of 12.6%, 34.6%, and 62.2% in low-, intermediate-, and high-risk patients, respectively. Notably, patients with score 0 had zero CLNM events, while those with score ≥4 exhibited >60% metastasis probability.

The four independent risk factors identified in our study are generally consistent with previous large cohort investigations, yet important discrepancies warrant critical examination. Wang et al. (11) demonstrated age <55 years as an independent predictor of CLNM in 8,668 PTMC patients (OR = 1.779, 95% CI: 1.569-2.017), whereas this variable failed to reach statistical significance in our multivariate analysis (adjusted OR = 1.31, 95% CI: 0.96-1.78, p=0.089). This divergence likely reflects population heterogeneity: our cohort included 29.2% patients with Hashimoto’s thyroiditis, an autoimmune condition that may modulate tumor microenvironment and attenuate age-related differences in biological aggressiveness (12). Furthermore, our preoperative ultrasound assessment—rather than relying solely on postoperative pathology (13)—may have captured more aggressive phenotypes, thereby diluting the independent effect of age. This finding suggests that in contemporary practice, where high-resolution ultrasound is universally available (14) and autoimmune thyroid disease is prevalent, risk stratification based solely on age thresholds may require reconsideration (15).

Tumor spatial location represents another dimension where our simplified approach diverges from contemporary frameworks. Sugitani et al. (16) have emphasized fine-grained spatial taxonomy—distinguishing anterior from posterior subcapsular and paratracheal locations—as critical for CLNM risk stratification, with recent modeling confirming that posterior-capsular and isthmic tumors carry higher risk when coded at this granularity (17). In our cohort, tumor location was categorized coarsely (upper/middle/lower/isthmus/diffuse) and showed no association with CLNM (p > 0.10), likely reflecting insufficient spatial resolution rather than a true absence of biological effect. Similarly, we did not routinely measure tumor-capsule distance (18) (19). These measurement differences highlight a familiar trade-off in PTMC risk modeling: fine-grained spatial data improve discrimination but require specialized radiological expertise, whereas bedside tools sacrifice spatial resolution for immediate usability. Our four-variable system follows the latter design philosophy.

The >5 mm cutoff demonstrated optimal discriminative performance (AUC 0.738) compared to alternative thresholds of 4 mm (AUC 0.712), 6 mm (AUC 0.721), and 8 mm (AUC 0.698). This finding aligns with the observation that CLNM risk increases continuously with tumor size (20), but our study further establishes 5 mm as the clinically optimal decision boundary. Notably, current guidelines suggest active surveillance consideration for unifocal intrathyroidal tumors ≤5 mm (21); our quantification of significantly elevated CLNM risk with tumors >5 mm (OR = 2.14) provides empirical support for this size threshold, consistent with recent meta-analytic evidence (10). Although this retrospective cohort was managed under the 2015 ATA guidelines (6), the scoring system’s risk stratification aligns with the 2025 ATA guidelines’ shift toward individualized, less routine dissection (7). The 2025 guidelines strongly discourage routine pCND for small, clinically node-negative PTCs, while conditionally supporting selective dissection in advanced local disease. Our system operationalizes this: score ≤1 (CLNM rate 12.6%) identifies patients suitable for active surveillance or limited surgery, whereas score ≥4 (CLNM rate 62.2%) meets criteria for selective pCND. However, we acknowledge that 5 mm represents a pragmatic rather than absolute biological boundary: sensitivity analyses demonstrated acceptable performance with a 6 mm threshold (AUC 0.721), supporting clinical flexibility to account for ultrasound measurement variability. An important finding was the zero CLNM rate among patients with score 0 (n=86), contrasting sharply with the 22.3% rate in score 1 patients. This suggests that score 0 identifies a distinct ‘very low-risk’ subgroup suitable for active surveillance without prophylactic surgery, consistent with Kuma hospital criteria for AS. Indeed, among the 86 score 0 patients (female, ≤5 mm, unifocal, no extrathyroidal extension), none had central lymph node metastasis, indicating that this profile may represent the ideal candidate for active surveillance strategies.

Several research groups have recently developed simplified PTMC risk assessment instruments. For instance, a nomogram combining sonographic findings and clinical factors achieved an AUC of 0.829 in predicting CLNM, incorporating variables such as age, multifocality, tumor size, microcalcification, and loss of fatty hilum (22). However, this model relies on complex ultrasound assessments including aspect ratio and C-TIRADS classification, which introduces inter-observer variability and requires specialized radiological expertise (23). Other sophisticated prediction models incorporating five or more variables have achieved superior discrimination (AUC >0.82), but their calculation requires electronic devices or nomogram charts, reducing bedside utility (24). Our scoring system offers distinct advantages: (1) exclusive dependence on four objective, preoperatively determinable variables eliminates subjective judgment variability; (2) integer weighting enables rapid mental calculation; (3) three risk tiers demonstrate clearly separated CLNM probabilities (12.6% vs. 34.6% vs. 62.2%) with substantial clinical separation. Although our AUC (0.738) is marginally lower than complex nomogram models, implementation science research consistently demonstrates that accessibility and usability often predict real-world clinical impact more accurately than statistical performance alone (25).

Machine learning approaches incorporating radiomic features have achieved higher statistical discrimination (26). However, machine learning models require standardized image acquisition protocols, specialized software, trained personnel, and computational infrastructure—requirements that create “implementation barriers” in resource-constrained settings. Furthermore, such models frequently exhibit performance degradation upon external validation (27), suggesting potential overfitting to specific equipment or populations. In contrast, our scoring system’s stable performance across sensitivity analyses and minimal resource requirements suggest superior real-world generalizability, acknowledging that machine learning remains preferable for tertiary centers with established platforms.

Our scoring system aligns with current guidelines’ principle of individualized surgical decision-making, but provides an operationalizable quantitative framework. Specific clinical applications are: Low-risk (0–1 points, CLNM rate 12.6%): meets low-risk criteria (unifocal, ≤5 mm, no extrathyroidal extension), suitable for active surveillance or lobectomy; Intermediate-risk (2–3 points, CLNM rate 34.6%): falls within the “gray zone,” where shared decision-making is appropriate; High-risk (≥4 points, CLNM rate 62.2%): exceeds low-risk thresholds, supporting prophylactic central neck dissection. Notably, among high-risk patients with clinically negative nodes on preoperative ultrasound, intraoperative frozen section confirmed CLNM in 58% of cases, supporting pCND even without sonographic suspicion.

All four variables are routinely assessed preoperatively without additional cost. We conducted preliminary usability testing with ten surgeons assessing hypothetical cases: use of the scoring system reduced assessment time and improved inter-rater agreement compared to conventional judgment. Although exploratory, this suggests simplified tools may improve decision efficiency. The minimal computational requirements offer value in settings lacking electronic health records. An important methodological consideration is that identical tumor characteristics may yield different risk tiers based on sex, as male gender contributes an independent point. This reflects its status as a significant predictor in our multivariate analysis (adjusted OR 1.68, 95% CI 1.12–2.53, p=0.013), consistent with meta-analytic evidence demonstrating higher CLNM odds in male PTMC patients (pooled OR 1.86, 95% CI 1.73–2.00) (28) and prior large cohort studies (11). Gender-stratified analyses from our cohort showed comparable discrimination in male (AUC 0.752, 95% CI 0.683–0.821) and female (AUC 0.731, 95% CI 0.679–0.783) subgroups, indicating the scoring system retains predictive utility within each sex stratum. Nevertheless, these thresholds were derived from the overall cohort and may not represent the optimal cutoffs for each gender individually. Clinicians should therefore interpret scores as population-level probability estimates rather than absolute individual risk, particularly when patient sex substantially influences the risk category. Future external validation studies should specifically assess whether sex-specific score thresholds improve calibration and clinical utility.

This study has several limitations. The single-center retrospective design and lack of BRAF V600E mutation data limit generalizability; sensitivity analysis excluding follicular variant PTC suggested modestly improved performance (AUC 0.751), indicating molecular markers could enhance future models. The parsimonious design prioritizes implementability over maximum accuracy—preoperative ultrasound features significant in univariate analysis were excluded, potentially misclassifying some high-risk patients (estimated false-negative rate ~13%). Clinicians should use this as a decision aid, not a replacement for judgment, especially with suspicious ultrasound features. We did not assess actual impact on surgical decisions; a stepped-wedge trial would be ideal to compare pCND rates and outcomes. Although the scoring system demonstrated good overall discrimination, the high-risk group (score ≥4) included only 74 patients (15.2%) with a CLNM rate of 62.2% (95% CI: 50.1-73.2%). The wide confidence interval reflects limited precision for this subgroup estimate. Clinicians should interpret the high-risk category as indicating substantial metastasis probability (>60%) rather than an exact quantitative risk, and external validation in larger cohorts is needed to refine these estimates. Finally, the minimal optimism correction (0.004) may underestimate true overfitting, underscoring the need for prospective external validation currently underway at two regional hospitals (target n=400).

## 5 Conclusions

This simplified four-variable scoring system effectively stratifies CLNM risk in PTMC patients using readily available preoperative variables. Low-risk patients (score ≤1) may be considered for less extensive surgery or active surveillance, whereas high-risk patients (score ≥4) should be considered for pCND. This practical tool enables immediate bedside risk assessment without specialized software; however, external validation is needed to confirm its generalizability across diverse clinical settings.

## Data Availability

The original contributions presented in the study are included in the article/**Supplementary Material**. Further inquiries can be directed to the corresponding author.

## References

[1] Siegel RL Miller KD Jemal A . Cancer statistics, 2020. Ca-Cancer J Clin. (2020) 70:7–30. doi: 10.3322/caac.21590 31912902

[2] Baloch Z Mete O Asa SL . Immunohistochemical biomarkers in thyroid pathology. Endocr Pathol. (2018) 29:91–112. doi: 10.1007/s12022-018-9532-9 29744727

[3] Davies L Welch HG . Current thyroid cancer trends in the United States. JAMA Otolaryngol. (2014) 140:317–22. doi: 10.1001/jamaoto.2014.1 24557566

[4] Schneider D Mazeh H Chen H Sippel R . Lymph node ratio predicts recurrence in papillary thyroid cancer. Oncologist. (2013) 18:157–62. doi: 10.1634/theoncologist.2012-0240 23345543 PMC3579599

[5] Lucandri G Fiori G Falbo F Pende V Farina M Mazzocchi P . Papillary thyroid microcarcinoma: Differences between lesions in incidental and nonincidental settings-considerations on these clinical entities and personal experience. Curr Oncol. (2024) 31:941–51. doi: 10.3390/curroncol31020070 38392064 PMC10888372

[6] Li XY Zhang B Lin YS . The interpretation of 2015 American Thyroid Association management guidelines for adult patients with thyroid nodules and differentiated thyroid cancer. Zhonghua Er Bi Yan Hou Tou Jing Wai Ke Za Zhi. (2017) 52:309–15. doi: 10.3760/cma.j.issn.1673-0860.2017.04.018 28441815

[7] Ringel MD Sosa JA Baloch Z Bischoff L Bloom G Brent GA . 2025 American Thyroid Association management guidelines for adult patients with differentiated thyroid cancer. Thyroid. (2025) 35:841–985. doi: 10.1177/10507256251363120 40844370 PMC13090833

[8] Ito Y Miyauchi A Inoue H Fukushima M Kihara M Higashiyama T . An observational trial for papillary thyroid microcarcinoma in Japanese patients. World J Surg. (2010) 34:28–35. doi: 10.1007/s00268-009-0303-0 20020290

[9] Li G Li R Zhong J Chen W Shuai J Chen M . A multicenter cohort study of thyroidectomy-related decision regret in patients with low-risk papillary thyroid microcarcinoma. Nat Commun. (2025) 16:2317. doi: 10.1038/s41467-025-57627-7 40057484 PMC11890561

[10] Wen X Jin Q Cen X Qiu M Wu Z . Clinicopathologic predictors of central lymph node metastases in clinical node-negative papillary thyroid microcarcinoma: a systematic review and meta-analysis. World J Surg Oncol. (2022) 20:106. doi: 10.1186/s12957-022-02573-7 35365171 PMC8976349

[11] Wang Y Guan Q Xiang J . Nomogram for predicting central lymph node metastasis in papillary thyroid microcarcinoma: A retrospective cohort study of 8668 patients. Int J Surg. (2018) 55:98–102. doi: 10.1016/j.ijsu.2018.05.023 29803769

[12] Cunha LL Morari EC Nonogaki S Bufalo NE da Assumpção LVM Soares FA . RORγt may influence the microenvironment of thyroid cancer predicting favorable prognosis. Sci Rep. (2020) 10:4142. doi: 10.1038/s41598-020-60280-3 32139737 PMC7058012

[13] Kim H Kim JA Son EJ Youk JH Chung TS Park CS . Preoperative prediction of the extrathyroidal extension of papillary thyroid carcinoma with ultrasonography versus MRI: a retrospective cohort study. Int J Surg. (2014) 12:544–8. doi: 10.1016/j.ijsu.2014.03.003 24631554

[14] Kwak JY Kim EK Youk JH Kim MJ Son EJ Choi SH . Extrathyroid extension of well-differentiated papillary thyroid microcarcinoma on US. Thyroid. (2008) 18:609–14. doi: 10.1089/thy.2007.0345 18578609

[15] Haugen BR Alexander EK Bible KC Doherty GM Mandel SJ Nikiforov YE . 2015 American Thyroid Association management guidelines for adult patients with thyroid nodules and differentiated thyroid cancer: The American Thyroid Association Guidelines Task Force on Thyroid Nodules and Differentiated Thyroid Cancer. Thyroid. (2016) 26:1–133. doi: 10.1089/thy.2015.0020 26462967 PMC4739132

[16] Sugitani I Ito Y Takeuchi D Nakayama H Masaki C Shindo H . Indications and strategy for active surveillance of adult low-risk papillary thyroid microcarcinoma: Consensus statements from the Japan Association of Endocrine Surgery Task Force on Management for Papillary Thyroid Microcarcinoma. Thyroid. (2021) 31:183–92. doi: 10.1089/thy.2020.0330 33023426 PMC7891203

[17] Pang N Cai R Zhang W Zhang R Sun Q Huang Y . An ultrasonographic tumor location-based model for predicting central lymph node metastasis in unifocal papillary thyroid microcarcinoma. Front Endocrinol. (2026) 17:1785660. doi: 10.3389/fendo.2026.1785660 42222094 PMC13215795

[18] Tian HY Yu ZY Dong T Xie Q Mu Y Liao W . Risk factors of cervical central lymph node metastasis in stage T1a unifocal papillary thyroid carcinoma. Sci Rep. (2024) 14:25577. doi: 10.1038/s41598-024-77681-3 39462054 PMC11513150

[19] Zhu M Zheng W Xiang Y Gu J Wang K Shang J . The relationship between central lymph node metastasis and the distance from tumor to thyroid capsule in papillary thyroid microcarcinoma without capsule invasion. Gland Surg. (2020) 9:727–36. doi: 10.21037/gs-20-478 32775263 PMC7347803

[20] Wang M Wu WD Chen GM Chou SL Dai XM Xu JM . Could tumor size be a predictor for papillary thyroid microcarcinoma: a retrospective cohort study. Asian Pac J Cancer P. (2015) 16:8625–8. doi: 10.7314/apjcp.2015.16.18.8625 26745127

[21] Haugen BR . 2015 American Thyroid Association management guidelines for adult patients with thyroid nodules and differentiated thyroid cancer: What is new and what has changed? Cancer-Am Cancer Soc. (2016) 123:372–81. doi: 10.1002/cncr.30360 27741354

[22] Duan S Yang Z Wei G Chen S Hu X Ryu YJ . Nomogram for predicting the risk of central lymph node metastasis in papillary thyroid microcarcinoma: a combination of sonographic findings and clinical factors. Gland Surg. (2024) 13:1016–30. doi: 10.21037/gs-24-154 39015718 PMC11247594

[23] Castellana M Castellana C Treglia G Giorgino F Giovanella L Russ G . Performance of five ultrasound risk stratification systems in selecting thyroid nodules for FNA. J Clin Endocr Metab. (2020) 105:1659–69. doi: 10.1210/clinem/dgz170 31690937

[24] Herrera D van de Veerdonk W Seibert D Boke M Gutiérrez-Ortiz C Yimer N . From algorithms to clinical utility: A systematic review of individualized risk prediction models for colorectal cancer. Gastrointest Disord (Basel). (2023) 5:549–79. doi: 10.3390/gidisord504004530654563

[25] Webster EM Perez L Ahsan MD Levi S Chandler I Thomas C . Integration and usability of a digital cancer risk stratification tool to optimize identification of patients at risk for hereditary cancers: A pilot study. Gynecol Oncol. (2024) 183:1–6. doi: 10.1016/j.ygyno.2024.02.028 38460222

[26] Liu X Li H Zhang L Gao Q Wang Y . Development and validation of a multidimensional machine learning-based nomogram for predicting central lymph node metastasis in papillary thyroid microcarcinoma. Gland Surg. (2025) 14:344–57. doi: 10.21037/gs-2024-508 40256479 PMC12004296

[27] Baishya N Baishya K . Integration of radiomics ultrasound and TIRADS in diagnosis of thyroid nodules: a narrative review. Egypt J Radiol Nucl Med. (2024) 55. doi: 10.1186/s43055-024-01381-138164791

[28] Orgoványi M Rancz A Dolhascu A Agócs G Szentes BL Sipter E . Risk factors associated with aggressive tumor phenotypes in papillary thyroid microcarcinoma: a systematic review and meta-analysis. Front Endocrinol. (2026) 17:1876912. doi: 10.3389/fendo.2026.1876912 42466333 PMC13372712

