# Supplementary material for: Risk factors for central lymph node metastasis in papillary thyroid microcarcinoma: a simplified risk scoring system
Source: Front Endocrinol (Lausanne). 2026 Sep 7;17:1833331. doi: 10.3389/fendo.2026.1833331 (PMC13590344; doi:10.3389/fendo.2026.1833331)
Supplement: Supplementary file 1 [file Table1.docx]

****Supplementary Table1**. Comparison of Scaling Factors for Risk Score Development**

| Scaling Factor | Score Range | ETE Points | Tumor >5mm Points | Male Points | Multifocality Points | AUC (95% CI) | Clinical Utility |
| --- | --- | --- | --- | --- | --- | --- | --- |
| ×1 (rounded) | 0–3 | 1 | 1 | 1 | 1 | 0.698 (0.653–0.743) | Poor (loses weight differentiation) |
| **×2 (final)** | **0–6** | **2** | **2** | **1** | **1** | **0.738 (0.693–0.783)** | **Optimal** |
| ×3 | 0–9 | 3 | 2 | 2 | 2 | 0.741 (0.696–0.786) | Overly complex |
| ×4 | 0–12 | 4 | 3 | 2 | 3 | 0.742 (0.697–0.787) | Excessive granularity |

×3 and ×4 scaling yield non-integer weights for tumor size (1.52→2) and multifocality (1.28→2), requiring rounding that distorts original β coefficients. AUC differences between ×2, ×3, and ×4 were minimal (<0.005), supporting selection of the simplest practical model.
